# Ineffective Degradation of Immunogenic Gluten Epitopes by Currently Available Digestive Enzyme Supplements

**DOI:** 10.1371/journal.pone.0128065

**Published:** 2015-06-01

**Authors:** George Janssen, Chantal Christis, Yvonne Kooy-Winkelaar, Luppo Edens, Drew Smith, Peter van Veelen, Frits Koning

**Affiliations:** 1 Department of Immunohematology and Blood Transfusion, Leiden University Medical Centre, Leiden, The Netherlands; 2 DSM Food Specialties, Delft, The Netherlands; 3 DSM Food Specialties, South Bend, United States of America; Tulane University, UNITED STATES

## Abstract

**Background:**

Due to the high proline content of gluten molecules, gastrointestinal proteases are unable to fully degrade them leaving large proline-rich gluten fragments intact, including an immunogenic 33-mer from α-gliadin and a 26-mer from γ-gliadin. These latter peptides can trigger pro-inflammatory T cell responses resulting in tissue remodeling, malnutrition and a variety of other complications. A strict lifelong gluten-free diet is currently the only available treatment to cope with gluten intolerance. Post-proline cutting enzymes have been shown to effectively degrade the immunogenic gluten peptides and have been proposed as oral supplements. Several existing digestive enzyme supplements also claim to aid in gluten degradation. Here we investigate the effectiveness of such existing enzyme supplements in comparison with a well characterized post-proline cutting enzyme, Prolyl EndoPeptidase from *Aspergillus niger* (AN-PEP).

**Methods:**

Five commercially available digestive enzyme supplements along with purified digestive enzymes were subjected to 1) enzyme assays and 2) mass spectrometric identification. Gluten epitope degradation was monitored by 1) R5 ELISA, 2) mass spectrometric analysis of the degradation products and 3) T cell proliferation assays.

**Findings:**

The digestive enzyme supplements showed comparable proteolytic activities with near neutral pH optima and modest gluten detoxification properties as determined by ELISA. Mass spectrometric analysis revealed the presence of many different enzymes including amylases and a variety of different proteases with aminopeptidase and carboxypeptidase activity. The enzyme supplements leave the nine immunogenic epitopes of the 26-mer and 33-mer gliadin fragments largely intact. In contrast, the pure enzyme AN-PEP effectively degraded all nine epitopes in the pH range of the stomach at much lower dose. T cell proliferation assays confirmed the mass spectrometric data.

**Conclusion:**

Currently available digestive enzyme supplements are ineffective in degrading immunogenic gluten epitopes.

## Introduction

Celiac disease (CD) is a chronic enteropathy caused by an uncontrolled immune response to wheat gluten and similar proteins of rye and barley in genetically susceptible individuals [1,2]. An important feature of the disease is characteristic flattening of intestinal villi along with crypt hypertrophy which results in a deleterious loss of mucosal surface for the efficient absorption of nutrients. If left untreated, celiac patients may suffer from complications including but not limited to growth retardation in children, nutritional insufficiencies, anemia, osteoporosis, infertility and neurological problems. At present, the only suitable treatment is a life-long exclusion of gluten from the patient’s diet.

CD only develops in individuals that express either HLA-DQ2 or HLA-DQ8 [1,2]. The molecular basis for this association is well understood: HLA-DQ2 and HLA-DQ8 bind particular (modified) gluten peptides and present these to pro-inflammatory T cells present in the small intestinal lamina propria of CD patients [1,2]. A prominent feature of gluten proteins is their high proline content [3]. Proline is the only amino acid whose side-group links to the α-amino group thereby complicating hydrolytic attack by proteases. Post-proline cleaving proteases exist in nature but are absent in the human gastric and pancreatic compartments so that relatively long proline-rich gluten fragments can reach the small intestine [4]. Here they bind to the disease predisposing HLA-DQ molecules and trigger pathogenic T cell responses. For optimal binding to HLA-DQ2 or HLA-DQ8 peptides must be at least nine amino acids long so that any enzyme that would degrade gluten proteins into smaller fragments would thereby destroy its disease inducing properties [1,2]. For this purpose oral supplementation with microbial proline-specific enzymes has been proposed [4]. Bacterial prolyl oligopeptidase from *Flavobacterium meningosepticum*, *Sphingomonas capsulate* and *Myxococcus xanthus* are indeed capable of breaking down toxic gluten sequences, but unfortunately their pH optimum is between 7 and 8 and thus outside the pH range of the stomach [5]. Moreover, such enzymes are effectively degraded by pepsin in the stomach [6]. More recently, ALV003, a combination of a cysteine protease present in barley and a prolyl endopeptidase from *Sphingomonas capsulata* was found to degrade gluten in the stomach [7,8]. Another combination, aspergillopepsin from *Aspergillus niger* and dipeptidyl peptidase IV from *Aspergillus oryzae*, was also effective in the detoxification of small doses of gluten *in vitro* [9]. Finally, we have investigated a novel type of prolyl endoprotease from the food grade fungus *Aspergillus niger* (AN-PEP) [6,10,11]. AN-PEP efficiently degrades gluten under the conditions mimicking the gastrointestinal tract [11] and was found to be safe both in animal studies and in humans [10,12]. Thus *in vitro* and *in vivo* experiments indicate that enzymes can be identified that degrade gluten proteins efficiently.

While the potential of post-proline cutting enzymes has now been well established, several other digestive enzyme blends are already marketed for gluten intolerance. Although these existing products incorporate complex proteolytic mixtures, proline-specific endoproteases are missing. In order to cope with the proline-rich gluten sequences such blends usually incorporate DPPIV, a fungal exopeptidase that can liberate X-Pro dipeptides from the amino-terminal side. On its own DPPIV has a very limited proteolytic effect as it can only act on proteins and peptides starting with X-Pro. Additionally, DPPIV has a neutral pH optimum so that it is unlikely to be active during stomach passage. Whether or not these commercial digestive blends are able to detoxify gluten is essentially unknown.

Here we have tested five commercially available digestive enzyme blends marketed for gluten intolerance using biochemical, immunological and mass spectrometric approaches. We show that, in contrast to AN-PEP, commercially available blends are completely ineffective in degrading immunogenic gluten epitopes.

## Materials and Methods

### Enzymes

Prolyl endopeptidase from *Aspergillus niger* (AN-PEP) was supplied by DSM Food Specialties, The Netherlands (lot DS70683). Dipeptidyl peptidase from *Aspergillus oryzae* (DPPIV) was chromatographically purified from Validase FP II, DSM Food Specialties (lot DS70103). To that end enzyme powder was dissolved in 20 mM sodium phosphate buffer pH 7.0, loaded onto a 50-ml Q-Sepharose column (GE Healthcare) and eluted using a 0 to 1M sodium chloride gradient. The resulting fractions were tested for DPPIV activity using the DAP enzyme assay with Ala-Pro-p-nitroanilide as a substrate. Fractions with the highest enzyme activity were pooled, concentrated and loaded onto on a 5-ml butyl-Sepharose HiTrap column (GE Healthcare) in a 50 mM sodium phosphate pH 7.3 buffer containing 1.5 M ammonium sulfate. The column was developed with a gradient from 1.5 M to 0 M ammonium sulfate in sodium phosphate pH 7.3. Fractions incorporating the highest DPPIV activity were pooled and concentrated. Human dipeptidyl peptidase was obtained from Prospec (Brunswick, NY; cat nr ENZ-375). Neutral pH leucine aminopeptidase (Corolase LAP) (lot DS70679) was obtained from AB Enzymes (Darmstadt, Germany).

Five different dietary supplements with gluten digesting label claims were commercially obtained and tested in this study (formulation given in S1 Text). These supplements are representative in formulation and characteristics for the US market as of the date the study commenced (2012). The selected supplements were from market leaders or contained ingredients from leading market ingredient suppliers such as DPPIV. The selected supplements contained the highest available DPPIV levels we could identify at the time the study commenced. The selection also covered the lowest and highest LAPU/DAP ratios available and one supplement (D) that mentions the presence of AN-PEP.

### Protein assay and SDS-PAGE

Protein concentration was determined by bicinchoninic acid protein assay (Thermo Scientific). SDS-PAGE was performed on pre-cast 4–12% acrylamide gradient gels using MES SDS buffer system (Life Technologies) in the presence of lithium dodecyl sulfate. Gels were stained with SimplyBlue SafeStain (Life Technologies). Selected bands were identified by mass spectrometry as detailed in S2 Text.

### Enzyme assays

Acid protease (HUT) assay is based on a 30-min proteolytic hydrolysis of denatured hemoglobin at pH 4.7 and 40°C and is carried out as described [13]. Non-hydrolyzed substrate is precipitated with trichloroacetic acid and removed by filtration. One Hemoglobin Unit on the Tyrosine basis (HUT) is defined as the amount of hydrolysate whose absorbance at 275 nm equals that of a solution containing 1.10 μg/ml tyrosine in 6 mM HCl.

Leucine aminopeptidase (LAP) activity is determined by the enzymatic hydrolysis of Leu-p-nitroanilide at pH 7.0 and 40°C. Resulting p-nitroaniline is determined spectrophotometrically at 405 nm. One Leucine AminoPeptidase Unit (LAPU) is defined as the amount of enzyme required to liberate one μmol of p-nitroaniline per minute [14].

X-prolyl dipeptidyl aminopeptidase (DAP) activity is based on the enzymatic hydrolysis at pH 7.5 and 30°C of Gly-Pro-p-nitroanilide into Gly-Pro and p-nitroaniline. Liberated p-nitroaniline is measured at 405 nm. One X-prolyl Dipeptidyl AminoPeptidase (DAP) unit is defined as the amount of enzyme required to liberate 1 μmol/min of p-nitroaniline [15]. AN-PEP was also tested in a modified DAP assay at pH 4.6 (instead of 7.5) and using N-carbobenzoxy-Gly-Pro-p-nitroanilide as a substrate.

### R5 ELISA

We used the competitive R5 ELISA immunoassay [16] (RIDASCREEN (R7021) and cocktail solution (R7006) from R-Biopharm) to circumvent the limitation of the sandwich ELISA which requires at least two epitopes in the protein or peptide to be detected. The R5 monoclonal antibody recognizes the gluten epitope QQPFP and related sequences commonly found in gluten and related proteins in barley and rye. Gliadin from wheat (Sigma, G3375) was dissolved in buffered 60% ethanol to prepare a concentrated stock solution. Content weight of dietary supplements was determined and buffered stock solutions were prepared at the equivalence of 0.08 capsule/ml. The reaction mixture consisted of 0.4 mg/ml gliadin, 0.2 mg/ml pepsin (Sigma, P6887), in 50 mM NaCl and either 100 mM sodium acetate pH 4.5 or 100 mM MES pH 6.2. This mixture was preheated to 37°C and the digestion reaction was started by adding supplement stock solution in the ratio of 1 g supplement per 0.5 g gliadin. Directly after adding the supplement and at 2, 5, 10, 15 and 30 minutes samples were taken. Reactions were stopped by cooling and increasing the pH to 11. Subsequently samples were treated according to manufacturer’s instructions with extraction cocktail (containing denaturing and reducing agents), before performing the ELISA procedure. The elaborate inactivation process was required in order to inactivate the proteases in the mixture, which would interfere with the ELISA readout system. In pilot experiments we observed that this was not effective for inactivation of the proteases in two supplements (D and E), presumably as they both contain subtilisins (see Results section), which is known to be stable and active at pH 11 [17]. Therefore these two supplements were excluded from our R5 ELISA analysis.

### Mass spectrometric analysis

The composition of digestive enzyme supplements was examined by nanoLC MS/MS as detailed in S2 Text. In short, supplements were purified by filter-aided sample preparation [18], followed by mass spectrometric identification of the constituting proteins.

Gluten epitope degradation was followed by on-line nano LC-electrospray mass spectrometry on a Q-TOF mass spectrometer (Micromass) as detailed in S2 Text. Reaction conditions for gluten degradation were optimized as detailed in S1 Fig. In short, reactions consisted of 40 μg peptide substrate (26-mer or 33-mer peptide) and digestive enzyme supplements (1–10 capsule equivalents) or AN-PEP (1/100-1 capsule equivalent) as control in a final volume of 200 μl 100 mM buffer pH 2.0 to 11.0 (see S2 Text for definition of capsule equivalents). Digestion was started by incubation at 37°C. After 0, 10, 30, 60 and 120 minutes, 40-μl aliquots were removed, chilled and processed as described in S2 Text. MS spectra were manually inspected for the presence of residual 26-mer or 33-mer and to identify peptides with m > 800 Da that potentially might contain gluten epitopes. Selected peptide products were subjected to MS/MS to identify gluten derived peptides.

### T cell proliferation assay

33-mer and its degradation products were deamidated using tissue transglutaminase (enzyme peptide ratio 1:5)(Sigma T5398), purified by C18 solid phase extraction and tested for T-cell activation. Proliferation assays were performed in triplicate in 150 μl IMDM supplemented with glutamine (Gibco, Life technologies) and 10% human serum in 96-well flat-bottom plates as described previously [19]. Briefly, antigen-presenting cells (APCs) were loaded with antigen (C18-purified gluten peptide and degradation products) for 2 h, after which 20,000 HLA-DQ2.5-glia-α-1 specific T cells were added. As APC’s we used 10^5^ irradiated HLA-DQ2-matched allogeneic PBMCs (3000 rad). After 48 h at 37°C, cultures were pulsed with 0.5 μCi of ^3^H-thymidine and harvested 18 hours later.

## Results

### Enzymatic activity of enzyme preparations

To measure enzyme activity in the supplements we used three commonly used assays: the HUT assay that determines broad spectrum endoproteolytic activity at acid pH, the LAP assay which measures aminopeptidase exoproteolytic cleavage after N-terminal leucine and the DAP/DPPIV assay that measures exoproteolytic cleavage after an N-terminal X-Pro sequence. Five commercially available dietary supplements marketed for gluten intolerance, indicated here as Supplement A, B, C, D and E, were selected and tested alongside three pure proteases (AN-PEP, DPPIV and LAP) for their HUT, LAP and DAP activities (Table 1). All enzyme supplements displayed comparable HUT, LAP and DAP activities (Table 1). High LAP activity was also observed for the pure Corolase LAP product. Although DPPIV is specifically mentioned on the label of all products, DAP activity in the supplements was very low in comparison with the purified DPPIV sample. As may be expected for a proline-specific enzyme, pure AN-PEP was ineffective in HUT or in LAP assay. In the HUT assay proteolytic activity is quantified by measuring the quantity of TCA-soluble peptides formed. Due to its proline-specificity, on a hemoglobin substrate AN-PEP generates rather large peptides of which only a minor fraction will be TCA-soluble. The LAP assay is carried out at neutral pH and measures cleavage of a Leu-pNA substrate. Therefore, it is not surprising that a proline-specific endoprotease with an acidic pH optimum shows no activity. AN-PEP also displayed moderate DAP activity, because the pH of the DAP assay (pH 7.5) is far outside the effective pH range of AN-PEP (pH 3–6) [6]; when tested at pH 4.6 the activity of AN-PEP was 45 PPU/g.

**Table 1:** Enzyme activities of digestive enzyme supplements and purified enzymes.

		Enzyme assay		
Digestive enzyme	Capsule content (mg)	HUT/mg (pH 4.7)	LAPU/g (pH 7.0)	DAP/g (pH 7.5)
Supplement A	695	47	67	1.09
Supplement B	335	210	273	0.60
Supplement C	465	210	293	0.55
Supplement D	395	290	335	0.78
Supplement E	425	81	76	0.27
AN-PEP (DSM)	275^#^	0.34	0	0.25*
DPPIV (DSM)		n.d.	n.d.	165
LAP (AB Enzymes)		0.063	378	0

The content of one capsule of supplement was weighed and the enzyme activities measured as specified in Materials and Methods. In the case of HUT, activity per capsule ((capsule content in mg) * (specific activity in HUT/mg)) matches with the product specification sheet within a factor three (see S1 Text)

^#^ AN-PEP is at present not commercially available in the form of a capsule; 275 mg is the intended capsule content

* AN-PEP was also tested in a modified DAP assay at pH 4.6 (instead of 7.5) and using N-carbobenzoxy-Gly-Pro-p-nitroanilide as a substrate. The specific activity of AN-PEP in this assay was 45 units/g.

n.d., not determined.

### Characterization of supplement composition

To obtain better insight in the composition of the supplements their protein composition was examined by mass spectrometry (Table 2). MS analysis identified amylases as the main constituents in all supplements and revealed the presence of several acid and alkaline proteases, but no DPPIV. Supplements D and E also contained subtilisins from bacterial origin.

Protein composition was also examined by SDS-PAGE followed by mass spectrometric analysis of selected protein bands. Supplements B, C and E showed comparable protein band patterns, suggesting a shared origin for these preparations (S2 Fig). MS analysis of the main band at 50 kDa confirmed that alpha and gluco amylases were major constituents of these three supplements next to a variety of proteases. The patterns of Supplements A and D were distinct from B, C and E, since these preparations showed no prominent protein bands larger than 20 kDa. AN-PEP displayed a single, major band corresponding to its molecular weight (66 kDa) (S2 Fig).

**Table 2:** Protein compositon of digestive enzyme supplements.

Identified Protein	Organism	Accession^1^	Suppl A	Suppl B	Suppl C	Suppl D	Suppl E
Gluco-amylase	*Aspergillus awamori*	AMYG_ASPAW	47	12		7	7
Alpha-amylase A type-3	*Aspergillus oryzae*	AMYA3_ASPOR	12	45	29	18	16
Alpha-amylase	*Bacillus amyloliquefaciens*	AMY_BACAM				9	
Beta-galactosidase^2^	*Aspergillus Niger*	BGAL_ASPNG	2		2		
Aspartic protease pep1	*Aspergillus oryzae*	PEPA_ASPOR	8	12	4		3
Aspartic protease pep1	*Aspergillus awamori*	PEPA_ASPAW	8				2
Alkaline protease 1	*Aspergillus oryzae*	ORYZ_ASPOR		6	6	2	
Subtilisin Carlsberg	*Bacillus licheniformis*	SUBT_BACLI				5	2
Subtilisin BPN	*Bacillus amyloliquefaciens*	SUBT_BACAM				3	

20 μg of digestive enzyme supplement was acid-inactivated, reduced, alkylated and digested with 2 μg of trypsin as specified in S2 Text Methods.Tryptic peptides were analyzed by LC-MS and the proteins identified using Mascot search engine. The numbers represent the number of unique peptides matching the protein.

^1^Uniprot database

^2^alternative name, Lactase-N

### Degradation of gliadin by digestive enzyme supplements as monitored by R5 ELISA immunoassay

Next we tested if the commercial enzyme preparations were capable of degrading gluten proteins under gastric conditions (corresponding with their presumed site of action). We incubated the enzyme supplements with gliadin for various periods at 37°C. We used a ratio of one capsule of supplement per 0.5 g of gliadin, the approximate amount of gliadin contained in one slice of bread. The reaction mixture further contained pepsin. We determined to what extent the enzyme preparations were able to eliminate the gliadin within 30 minutes. This interval was chosen as gastric emptying half time is about 1h [20], which yields an estimated 25% of the gastric content reaching the duodenum in 30 minutes. Therefore gluten should be eliminated rapidly. As a readout system we used the R5 monoclonal antibody based ELISA, accepted by the Codex Alimentarius Commission (www.codexalimentarius.org) (CODEX STAN 118–1979) for determining gluten content in foods [16]. The FDA set a gluten limit of less than 20 ppm in gluten-free foods (www.fda.gov/ForConsumers)[21], which requires a degradation of more than 99.9% of the 0.5 g gliadin to be safe for the gluten intolerant patient. We first determined the pH optimum of the enzyme supplements and of AN-PEP on a model peptide. In agreement with previous results [6], AN-PEP was found to be active at pH between 3 and 6 while the enzyme supplements were inactive at pH 5 and lower, but fully active at pH 6–8 (S3 Fig). Gliadin degradation was therefore tested at both pH 4.5 and 6.2. Supplements D and E were excluded from our R5 ELISA analysis because their composition was incompatible with the ELISA.

**Fig 1:**
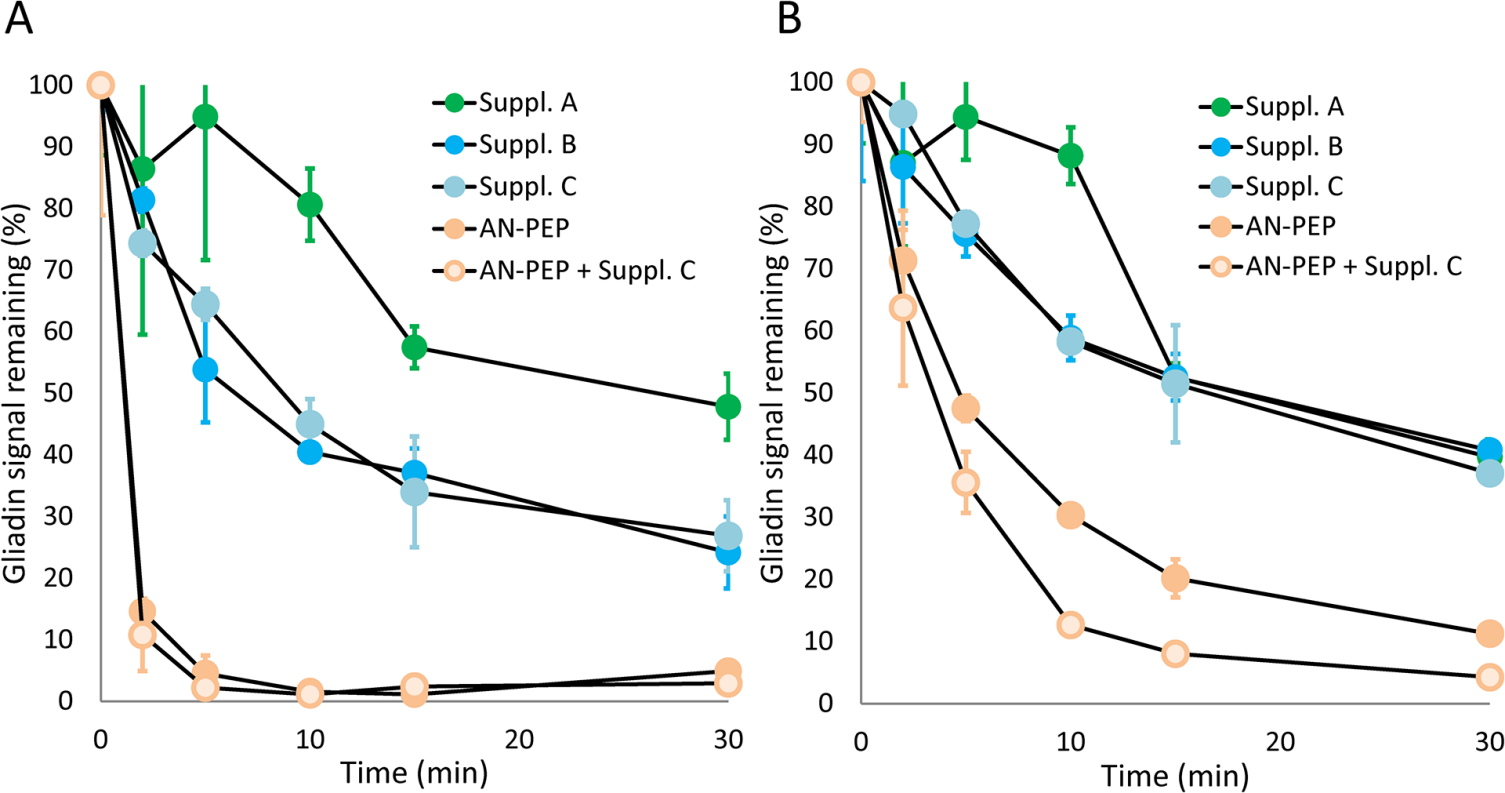
Degradation of gliadin as monitored by R5 ELISA immunoassay. Degradation of gliadin was monitored as a function of time at pH 4.5 (A) and pH 6.2 (B), using AN-PEP as a control enzyme. To exclude the presence of an interfering substance in digestive enzyme supplements we also analyzed AN-PEP control enzyme in the presence of Supplement C. Error bars represent standard deviation of duplicate experiments. Supplements D and E were not tested since they were not compatible with R5 ELISA immunoassay procedure.

While AN-PEP completely removed immune-reactive peptides already within a 10 min incubation period at pH 4.5, the supplements were unable to completely degrade R5 reactive peptides at either pH 4.5 or 6.2, (Fig 1A and 1B). AN-PEP was less active at an elevated pH of 6.2 although it still outperformed the tested supplements. To exclude that one or more substances in the enzyme supplements interfered with our readout we also analyzed the effect of enzyme supplements on the activity of AN-PEP. We did not observe such interference at either pH. From the degradation kinetics at pH 4.5, we conclude that, relative to AN-PEP, the digestive enzyme supplements showed modest gliadin degradation capacity (Fig 1A). However, after 30 minutes of incubation at pH 4.5, the enzyme supplements were able to degrade 50–70% of the gliadins.

### Mass spectrometric analysis of the degradation of gluten epitopes

Although the results with the R5 ELISA indicate that the supplements are able to substantially degrade gliadin, this does not implicate that immunogenic gluten epitopes are degraded since many toxic gluten epitopes do not contain the QQPFP sequence detected by the R5 ELISA. Therefore we used an alternative approach to reliably monitor the breakdown of well characterized immunogenic sequences of gluten through mass spectrometry. In particular we studied the breakdown of synthetic peptides corresponding to the α-gliadin derived 33-mer and the γ-gliadin derived 26-mer that arise from breakdown of gluten in the gastrointestinal tract [4,22,23]. The 33-mer contains six copies corresponding to the immunodominant DQ2.5-glia-α1 and DQ2.5-glia-α2 epitopes while the 26-mer contains the immunogenic DQ2.5-glia-γ3, DQ2.5-glia-γ4c and DQ2.5-glia-γ5 epitopes (Table 3).

**Table 3:** Epitopes contained in the gastrointestinal-enzyme resistant 26-mer and 33-mer gluten peptides.

**26-mer**	**FLQPQQPFPQQPQQPYPQQPQQPFPQ**
**DQ2.5-glia-γ5**	**----QQPFPQQPQ-------------**
DQ2.5-glia-γ3^*^	**---------QQPQQPYPQ--------**
DQ2.5-glia-γ4c^*^	**-----------------QQPQQPFPQ**
**33-mer**	**LQLQPFPQPQLPYPQPQLPYPQPQLPYPQPQPF**
**DQ2.5-glia-α1a**	**----PFPQPQLPY--------------------**
**DQ2.5-glia-α1b**	**-----------PYPQPQLPY-------------**
**DQ2.5-glia-α1b**	**------------------PYPQPQLPY------**
**DQ2.5-glia-α2**	**------PQPQLPYPQ------------------**
**DQ2.5-glia-α2**	**-------------PQPQLPYPQ-----------**
**DQ2.5-glia-α2**	**--------------------PQPQLPYPQ----**

The sequences of the 26-mer and 33-mer peptide are shown. Underneath, the position of the immunogenic epitopes is denoted

^*^the epitopes DQ8-glia-γ1a and DQ8-glia-γ1b are identical to epitopes DQ2.5-glia-γ4c respectively DQ2.5-glia-γ3

**Fig 2:**
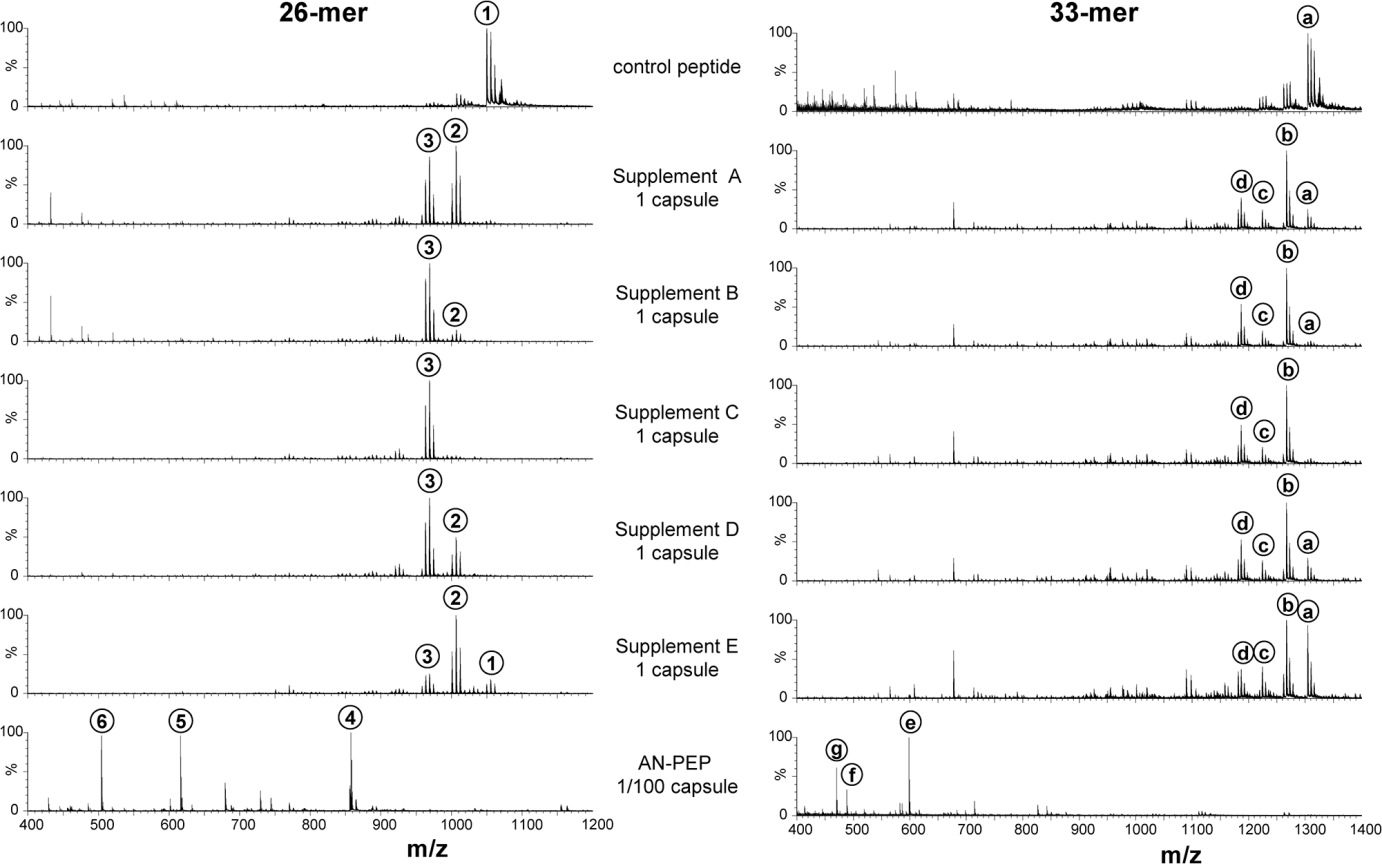
Mass spectrometric analysis of the degradation products of 26-mer and 33-mer peptide by digestive enzyme supplements. Peptide was incubated at 37°C for 30 minutes at pH 6.0 in the presence of 1 capsule equivalent digestive enzyme supplements. As a control, 1/100 capsule equivalent of AN-PEP at pH 5.0 was also incubated. Peptide reaction products were analyzed by Q-TOF LC-MS and their MS spectra shown in this Figure. The identity of the prominent peptides 1–6 and a-g are given in Table 4. In the case of AN-PEP, small amounts of epitope-containing peptides were observed under these conditions, but they disappear at higher enzyme concentration or prolonged incubation (see Fig 3). The digestive enzyme supplements display comparable activities and remove at most two amino acids from the N-terminus of the 26-mer (peptide 1 (26-mer) is degraded to peptide 2, and then to peptide 3), and three amino acids at most from the 33-mer peptide (peptide a (33-mer) is degraded to b, to c, and then to peptide d).

The degradation products of the 26-mer and 33-mer were investigated at all pHs between 2.0 and 11.0 for the five digestive enzyme supplements and AN-PEP (Fig 2, and S4 and S5 Figs). A comparison of the peptide mass spectrum after degradation at pH 5.0 for AN-PEP, and pH 6.0 for the supplements is shown in Fig 2, with annotation as detailed in Table 4. Under these conditions, the original 26-mer (m/z 1049.67 (3+)) and 33-mer (m/z 1304.27 (3+)) peptides were degraded to lower mass peptide species by all enzymes. However, analysis of the resulting new peptide species indicated that the enzyme supplements only removed a few N-terminal amino acids from the 26- and 33-mer, thus leaving nine immunogenic sequences intact (Table 4). In contrast and in agreement with previous results [6,11], with AN-PEP the large majority of resulting peptides were eight amino acids long or smaller and thus too small to still contain immunogenic epitopes.

**Table 4:** Peptides generated from the 26-mer and 33-mer.

**26-mer and derived peptides**		
	**m/z**	**peptide**
**1**	**1049.67(3+)**	**FLQPQQPFPQQPQQPYPQQPQQPFPQ**
**2**	**1001.19(3+)**	**-LQPQQPFPQQPQQPYPQQPQQPFPQ**
**3**	**963.83(3+)**	**--QPQQPFPQQPQQPYPQQPQQPFPQ**
**4**	**857.53(1+)**	< 8 amino acids*
**5**	**616.37(1+)**	< 8 amino acids*
**6**	**504.33(1+)**	< 8 amino acids*
**33-mer and derived peptides**		
	**m/z**	**peptide**
	**1304.27(3+)**	**LQLQPFPQPQLPYPQPQLPYPQPQLPYPQPQPF**
**b**	**1266.42(3+)**	**-QLQPFPQPQLPYPQPQLPYPQPQLPYPQPQPF**
**c**	**1223.75(3+)**	**--LQPFPQPQLPYPQPQLPYPQPQLPYPQPQPF**
**d**	**1186.03(3+)**	**---QPFPQPQLPYPQPQLPYPQPQLPYPQPQPF**
**e**	**598.46(1+)**	< 8 amino acids*
**f**	**488.34(1+)**	< 8 amino acids*
**g**	**470.39(1+)**	< 8 amino acids*

Numbers and letters refer to peaks in Fig 2

Sequences that contain epitopes are underlined

*peptides shorter than eight amino acids are considered too short to contain an epitope

**Fig 3:**
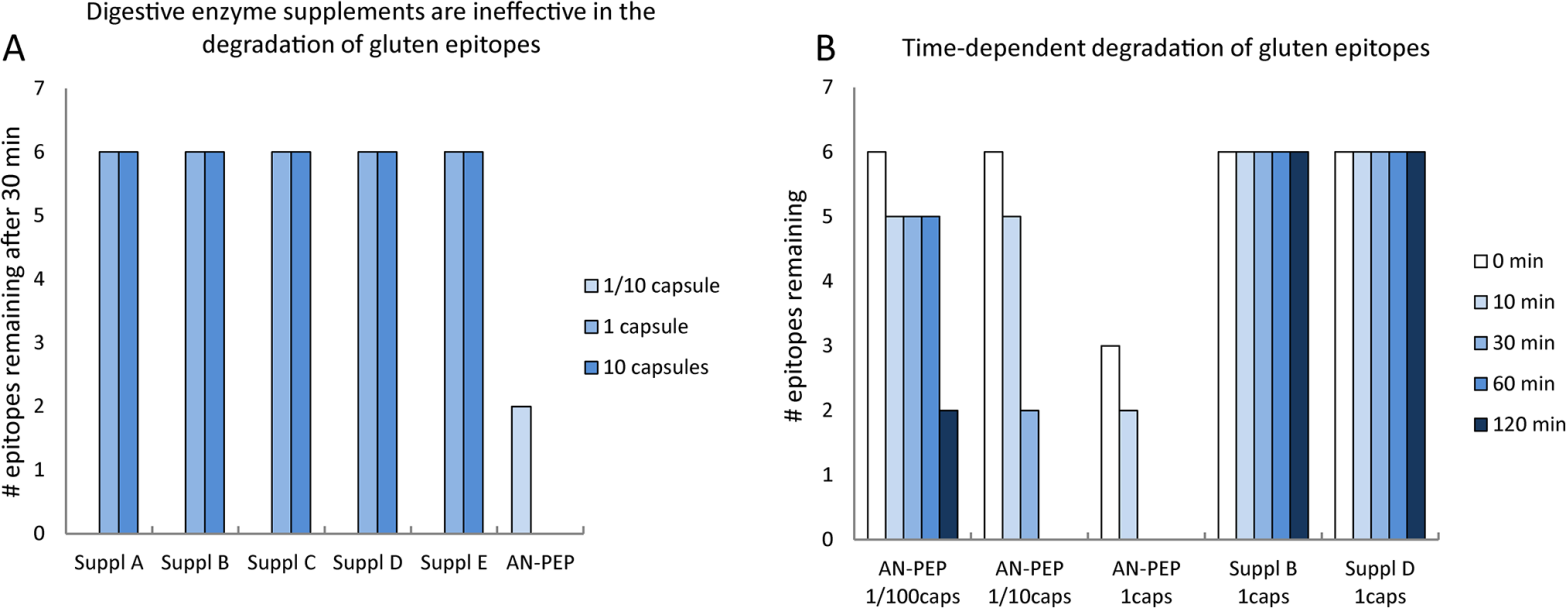
Digestive enzyme supplements do not neutralize any of the 6 gluten epitopes of the 33-mer, whereas the control enzyme AN-PEP does. The chart shows the number of epitopes found in the reaction mixture after incubation with digestive enzyme supplements or AN-PEP. An epitope is counted as present irrespective of its concentration, or the form in which it appears, 33-mer or one of its degradation products. Reactions with digestive enzymes were performed at their optimal pH 7.0, and reactions with AN-PEP at pH 5.0. In addition, when tested in the whole pH range 2 to 11, none of the digestive enzyme supplements was able to neutralize a single epitope (see also S4 and S5 Figs). A, No neutralization at a dose of 1 or 10 capsule equivalents digestive enzyme supplements (1/10 capsule was not tested) after 30 minutes at 37°C. As a control, AN-PEP was tested at a dose of 1/10 and 1 capsule. B, No neutralization of epitopes by digestive enzyme supplements, even after 120 minutes of incubation at 37°C. As a control, the time-dependent degradation by AN-PEP is shown.

Strikingly, the digestive enzyme supplements all showed very comparable degradation profiles (Fig 2). All supplements displayed the same aminopeptidase exoprotease activity yielding shortened 26-mer products lacking one or two amino terminal amino acids, 26-mer-F (m/z 1001.19 (3+)) and 26-mer-FL (m/z 963.83 (3+)) (Fig 2 and Table 4). The removal of the subsequent QP dipeptide, which may be performed by DPPIV activity, was not observed with supplements while purified DPPIV enzymes from *Aspergillus* and from human origin were able to remove amino terminal QP or FP dipeptides in our assay system (results not shown). At an elevated dose of 10 capsule equivalents an additional carboxypeptidase exoprotease activity was found active in the pH 3.0–5.0 range (see S2 Text for definition of capsule equivalents). This activity removes the carboxy-terminal Q and thereby partially neutralizes the DQ2.5-glia-γ4c epitope of the 26-mer (results not shown). Similarly, all enzyme supplements (1 capsule equivalent) removed one-to-three amino acids from the amino terminus of the 33-mer but left all six epitopes contained in the 33-mer intact, 33-mer-L (m/z 1266.42 (3+)), 33-mer-LQ (m/z 1223.75 (3+)) and 33-mer-LQL (m/z 1186.03 (3+)). In summary, under no conditions did the commercial enzyme supplements yield degradation products of the 26- and 33-mer that were devoid of immunogenic sequences. This is illustrated in Fig 3 where a qualitative summation of the number of epitopes after reaction with digestive enzyme supplements is presented. Thus, as long as the complete epitope (as defined in Table 3) is found by mass spectrometry, it is counted as 1, irrespective of its concentration or the form in which it appears, since for a patient, even small amounts of an epitope may be harmful. Experiments with digestive enzyme supplements have been repeated under different pH conditions and doses, all with the same outcome: all epitopes remain present (S4 and S5 Figs).

### Ineffective detoxification of the 33-mer as determined by T cell proliferation

**Fig 4:**
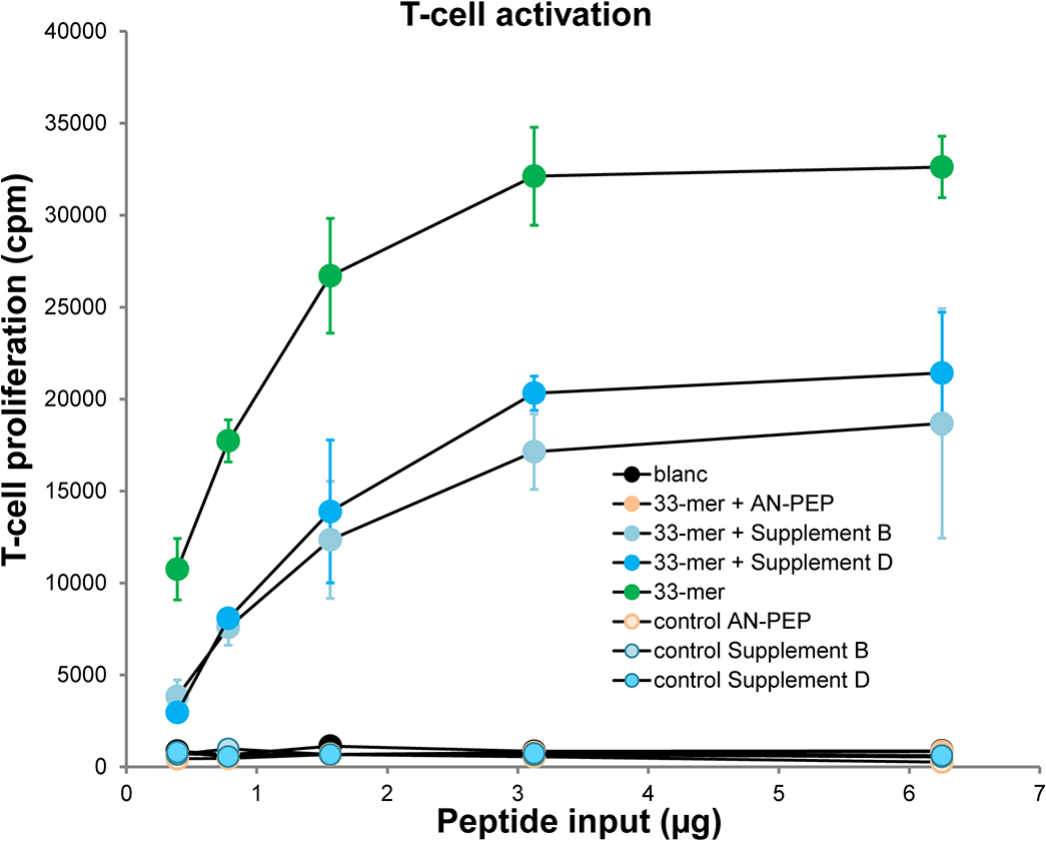
Ineffective detoxification by digestive enzyme supplements. 33-mer was incubated for 60 minutes with 1 capsule equivalent of digestive enzyme supplements, or 1/10 capsule equivalent of AN-PEP as a control. The peptide reaction products were deamidated using tissue transglutaminase, purified by C18 solid phase extraction and tested for T-cell activation. As a negative control, T-cells were incubated without the 33-mer peptide or digestive enzyme supplements (blanc). As a positive control, T-cells were incubated with 33-mer peptide. The enzyme preparations, when tested separately, were not toxic for the T-cells (not shown). T-cells stimulated with control peptide (LQLQPFPQPELPYPQPELPYPQPELPYPQPQPF) incorporated 40,000 cpm. T-cell proliferation ([^3^H]thymidine incorporation in cpm) is shown as a function of the input of 33-mer and its degradation products. Error bars represent standard deviation of triplicate experiments. The result shown is representative for two independent experiments.

To verify that the commercial enzyme supplements cannot neutralize the immunogenic properties of gluten, we tested a gluten-specific T cell clone against enzyme treated 33-mer. We used T cell clone N10 specific for the DQ2-glia-α1 epitope that is present in three copies in the 33-mer (Table 3) [4,24]. 33-mer was treated for 60 min with a one capsule dose of either Supplement B or D or a 1/10 capsule dose of AN-PEP. The degradation products were added to T cells and antigen presenting cells expressing the appropriate HLA-DQ2 molecules. Supplements B and D were chosen as representatives for the two groups according to SDS-PAGE, S2 Fig. As controls the enzyme preparations as such and untreated 33-mer were included. After 48 hours the specific T cell proliferation was measured. The results demonstrate that, unlike AN-PEP, the two commercial enzyme preparations were unable to neutralize the T cell stimulatory properties of the 33-mer (Fig 4).

## Discussion

A lifelong gluten-free diet is currently the only available treatment for gluten intolerant individuals. Although effective, such a diet is difficult to maintain due the frequent usage of gluten in the food industry. On average a person consumes between 15 and 20 grams of gluten daily while on a normal diet, most of which is derived from food products that are commonly associated with wheat like bread, cookies, pizza and pasta. However, some of the gluten comes from other sources as it is often a hidden ingredient and naturally gluten-free products can contain (traces of) gluten due to contamination during cultivation, transport and manufacturing of food products. Thus, additional approaches to protect against inadvertent gluten consumption are highly desired. The gluten and gluten-like proteins from wheat, barley and rye have an unusual high proline content. Due to the specific properties of proline, proteins rich in proline resist proteolytic degradation. As a result, long gluten fragments escape further degradation by pepsin in the stomach and by trypsin and chymotrypsin in the small intestine. Resulting fragments can bind to the predisposing HLA-DQ2 and HLA-DQ8 molecules and trigger pro-inflammatory T cell responses in patients with CD.

Several companies sell enzyme supplements marketed as digestive support for a gluten-free life style with highly suggestive brand names. These supplements could thus easily entice patients to try these supplements to counteract the deleterious effects of (inadvertent) gluten consumption. Here we investigated the composition of such enzyme supplements and tested their ability to degrade gluten proteins and immunogenic fragments thereof. We observed that the enzyme preparations indeed contain proteolytic activity but that the major constituents of the preparations are amylases. We observed that the pH optimum of the principal proteolytic activity in the enzyme supplements is between pH 6.0 and 8.0 and thus outside the pH range of the stomach. Moreover, we found that the proteolytic activity of the preparations could only partly neutralize gluten proteins as determined by ELISA. In this respect it is important that while the R5 ELISA used is approved by the Codex Alimentarius Commission for determining gluten content in foods, it is specific for a peptide sequence (QQPFP) that is not present in the immunodominant sequences from α-gliadins but is present in a number of γ-gliadin sequences. Thus, the observed reduction in gluten content with the enzyme supplements as determined with the R5 ELISA is not likely to reflect an actual breakdown of all immunogenic sequences. This is corroborated by mass spectrometric analyses of gluten degradation products which indicates that the commercial enzyme supplements were completely ineffective in degrading immunogenic gluten fragments from both the α- and γ-gliadins. We would like to stress that we have tested the enzyme supplements under a variety of pH conditions and that we have used state of the art mass spectrometry to follow the degradation of well characterized immunogenic α- and γ-gliadin fragments. These 33-mer and 26-mer fragments have been shown to arise from gastrointestinal degradation of gliadin proteins [4,22]. It is well established that the 33-mer fragment contains six copies of immunodominant gluten peptides to which T cell responses are almost invariably found in HLA-DQ2 positive patients. Similarly, the 26-mer fragment contains three immunogenic peptides. Mass spectrometry is a very sensitive technique that allows highly accurate and sensitive detection and identification of peptides and degradation products thereof. Our results unequivocally demonstrate that the enzyme supplements are only capable of removing a few N-terminal amino acids from both the 33- en 26-mer gliadin fragments, leaving the nine immunogenic epitopes intact. Only at an elevated dose of 10 capsule equivalents we observed that a single epitope of the 26-mer was partially degraded. Thus, the currently available enzyme supplements cannot be used to counteract the effect of gluten consumption in gluten intolerant individuals. Importantly, this implies that all enzyme preparations with a similar composition will fail to effectively degrade gluten.

Finally, next to CD, there is presently much attention for so-called non-celiac gluten sensitivity [25]. Although this is at present an ill-defined disease entity, it has led to the notion that gluten containing cereals pose a general risk to health. Evidently, our observation that the commercial enzyme supplements tested here do not degrade gluten indicates that they would neither be beneficial in this type of disorder.

## Supporting Information

S1 Fig Optimization of reaction conditions using pure AN-PEP. A, First the optimal amount of AN-PEP was assessed by a 30 minutes incubation with 26-mer at pH 5.0. It is seen that approximately 100 ng of AN-PEP is sufficient to degrade almost all of the peptide. B, With this amount, the optimal time range was verified with 100 ng (closed circles) and without AN-PEP (open circles). Thus, 100 ng of ANPEP and 30 minutes incubation was found to be optimal for the degradation of ~90% of the 26-mer substrate. Error bars in A and B represent standard deviation for triplicate measurements.
(PDF)

S2 Fig SDS PAGE analysis of digestive enzyme supplements and AN-PEP. 20 ug of digestive enzyme supplement or AN-PEP was denatured, boiled in SDS sample buffer (reducing) and applied on the 4–12% gel. The gel was stained with Coomassie blue (SafeStain). Selected protein bands (asterisk) were analyzed by mass spectrometry (see S2 Text for Method)and turned out to be amylase. Note that the supplements might be divided into two groups: Supplements B, C and E have very similar protein band composition. Supplements A and D also show similarities.
(PDF)

S3 Fig Determination of pH optimum of digestive enzyme supplements using 1 capsule equivalent (A) and AN-PEP using 1/100 capsule equivalent as control (B). Supplements were incubated for 30 minutes at all the pHs 2 to 11 with 26-mer gluten peptide. The decrease of intact 26-mer (m/z 1049 (3+)) was monitored by mass spectrometry and taken as measure for activity. Note that in the case of supplements, the decrease of 26-mer does not reflect degradation of epitopes, but only exoprotease activity removing one to two amino acids at most from the N-terminus (Fig 2, and S4 and S5 Figs). Error bars in A and B represent standard deviation for triplicate measurements.
(PDF)

S4 Fig Degradation of 26-mer by AN-PEP and digestive enzyme supplement. 26-mer peptide was incubated for 30 min with 1/100 capsule equivalent of AN-PEP (A) or with 1 capsule equivalent of Supplement A (B) at 10 different pHs 2–11. **A,** The peptides with m/z 504 (1+), 616 (1+) and 857 (1+) are short breakdown products derived from the 26-mer; a peptide with less than 8 amino acids is too short to contain an epitope. The small amounts of 679 (2+) and 1154 (2+) peptides disappear after prolonged treatment. **B,** The peptides with m/z 963 (3+) and 1001 (3+) still contain all three epitopes of the 26-mer 1049 (3+). Degradation patterns of the other 4 enzyme supplements are very similar to that of Supplement A. Epitope-containing peptides are marked with a dot (epitopes are underlined). Because of ammonia adduct formation, peptides resolve into several closely spaced peaks. The m/z values in the spectra correspond to the most intense species; the m/z shown below correspond to the monoisotopic mass.
(PDF)

S5 Fig Degradation of 33-mer by digestive enzyme supplement D and AN-PEP. 33-mer peptide was incubated for 30 min with 1 capsule equivalent of digestive enzyme supplement or 1/100 capsule equivalent of AN-PEP at pH 2.0, 5.0 and 7.0 as shown. Peptides were analyzed by LC-MS as detailed in S2 Text Methods. Peptides containing epitopes are marked with a dot. The small residual amounts of 33-mer and 33-mer-LQLQP m/z 1111 (3+) in the case of AN-PEP disappear after prolonged treatment. Because of ammonia adduct formation, peptides resolve into several closely spaced peaks (see also S4 Fig). Degradation patterns of the other 4 digestive enzyme supplements are very similar to that of Supplement D.
(PDF)

S1 Table Calculation of capsule equivalents. *AN-PEP is at present not commercially available in the form of a capsule; 275 mg is the intended capsule content; an amount of 100 ng AN-PEP compares to 1/100 capsule equivalent in the downscaled (27,500 x) assay. Capsule equivalents for digestive enzyme supplements were corrected for capsule content using a x b/c, where a = 100 ng, the optimal amount AN-PEP; b is the capsule content of enzyme supplements in mg; and c = 275 mg, the intended capsule content for AN-PEP
(PDF)

S1 Text Formulation of selected enzyme supplements. (PDF)

S2 Text Supporting Information Methods. (PDF)
