# Supplementary material for: Ineffective Degradation of Immunogenic Gluten Epitopes by Currently Available Digestive Enzyme Supplements
Source: PLoS One. 2015 Jun 1;10(6):e0128065. doi: 10.1371/journal.pone.0128065 (PMC4452362; doi:10.1371/journal.pone.0128065)
Supplement: S1 Fig — A, First the optimal amount of AN-PEP was assessed by a 30 minutes incubation with 26-mer at pH 5.0. It is seen that approximately 100 ng of AN-PEP is sufficient to degrade almost all of the peptide. B, With this amount, the optimal time range was verified with 100 ng (closed circles) and without AN-PEP (open circles). Thus, 100 ng of ANPEP and 30 minutes incubation was found to be optimal for the degradation of ~90% of the 26-mer substrate. Error bars in A and B represent standard deviation for triplicate measurements. (PDF) [file pone.0128065.s001.pdf]

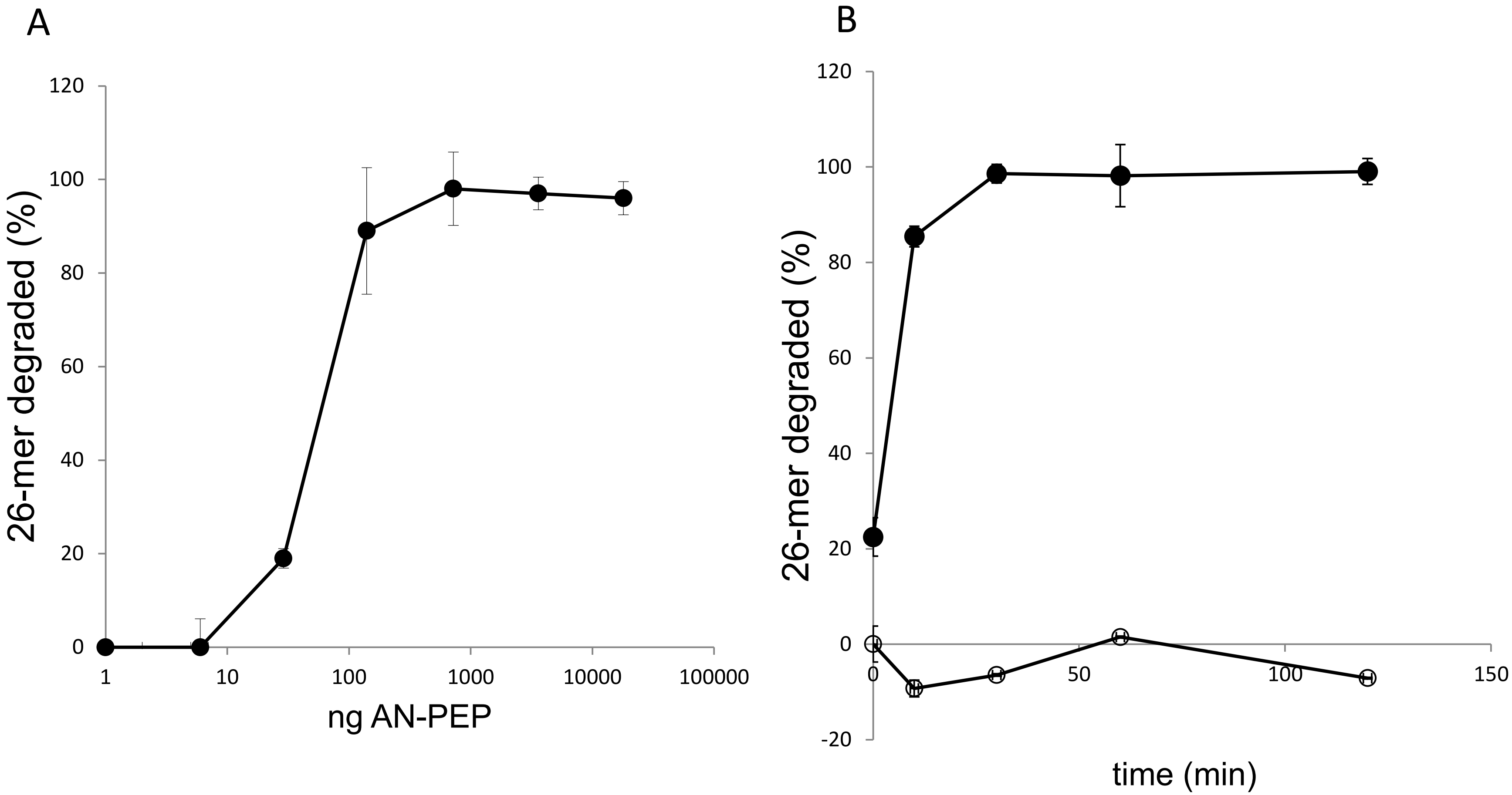

S1 Fig. Optimization of reaction conditions using pure AN-PEP. A, First the optimal amount of AN-PEP was assessed by a 30 minutes incubation with 26-mer at pH 5.0. It is seen that approximately 100 ng of AN-PEP is sufficient to degrade almost all of the peptide. B, With this amount, the optimal time range was verified with 100 ng (closed circles) and without AN-PEP (open circles). Thus, 100 ng of ANPEP and 30 minutes incubation was found to be optimal for the degradation of ~90% of the 26-mer substrate. Error bars in A and B represent standard deviation for triplicate measurements.
