# Supplementary material for: Ineffective Degradation of Immunogenic Gluten Epitopes by Currently Available Digestive Enzyme Supplements
Source: PLoS One. 2015 Jun 1;10(6):e0128065. doi: 10.1371/journal.pone.0128065 (PMC4452362; doi:10.1371/journal.pone.0128065)
Supplement: S2 Fig — 20 ug of digestive enzyme supplement or AN-PEP was denatured, boiled in SDS sample buffer (reducing) and applied on the 4–12% gel. The gel was stained with Coomassie blue (SafeStain). Selected protein bands (asterisk) were analyzed by mass spectrometry (see S2 Text for Method)and turned out to be amylase. Note that the supplements might be divided into two groups: Supplements B, C and E have very similar protein band composition. Supplements A and D also show similarities. (PDF) [file pone.0128065.s002.pdf]

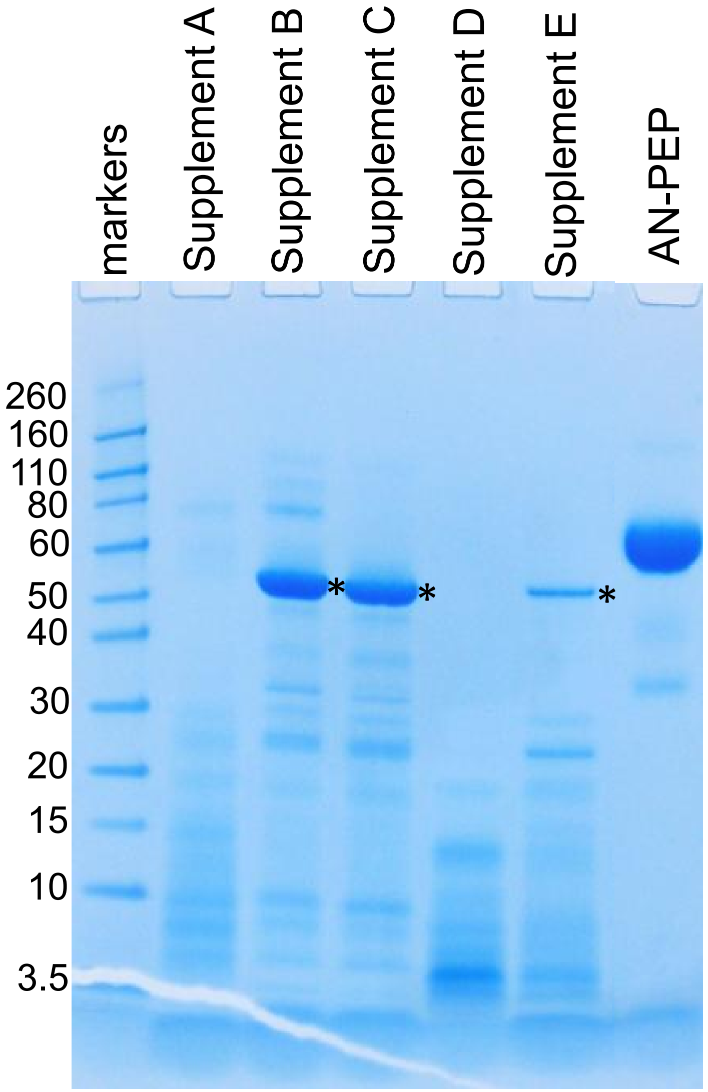

S2 Fig. SDS PAGE analysis of digestive enzyme supplements and AN-PEP. 20 ug of digestive enzyme supplement or AN-PEP was denatured, boiled in SDS sample buffer (reducing) and applied on the 4-12% gel. The gel was stained with Coomassie blue (SafeStain). Selected protein bands (asterisk) were analyzed by mass spectrometry (see S2 Text for Method) and turned out to be amylase. Note that the supplements might be divided into two groups: Supplements B, C and E have very similar protein band composition. Supplements A and D also show similarities.
