# Supplementary material for: Ineffective Degradation of Immunogenic Gluten Epitopes by Currently Available Digestive Enzyme Supplements
Source: PLoS One. 2015 Jun 1;10(6):e0128065. doi: 10.1371/journal.pone.0128065 (PMC4452362; doi:10.1371/journal.pone.0128065)
Supplement: S3 Fig — Supplements were incubated for 30 minutes at all the pHs 2 to 11 with 26-mer gluten peptide. The decrease of intact 26-mer (m/z 1049 (3+)) was monitored by mass spectrometry and taken as measure for activity. Note that in the case of supplements, the decrease of 26-mer does not reflect degradation of epitopes, but only exoprotease activity removing one to two amino acids at most from the N-terminus (Fig 2, and S4 and S5 Figs). Error bars in A and B represent standard deviation for triplicate measurements. (PDF) [file pone.0128065.s003.pdf]

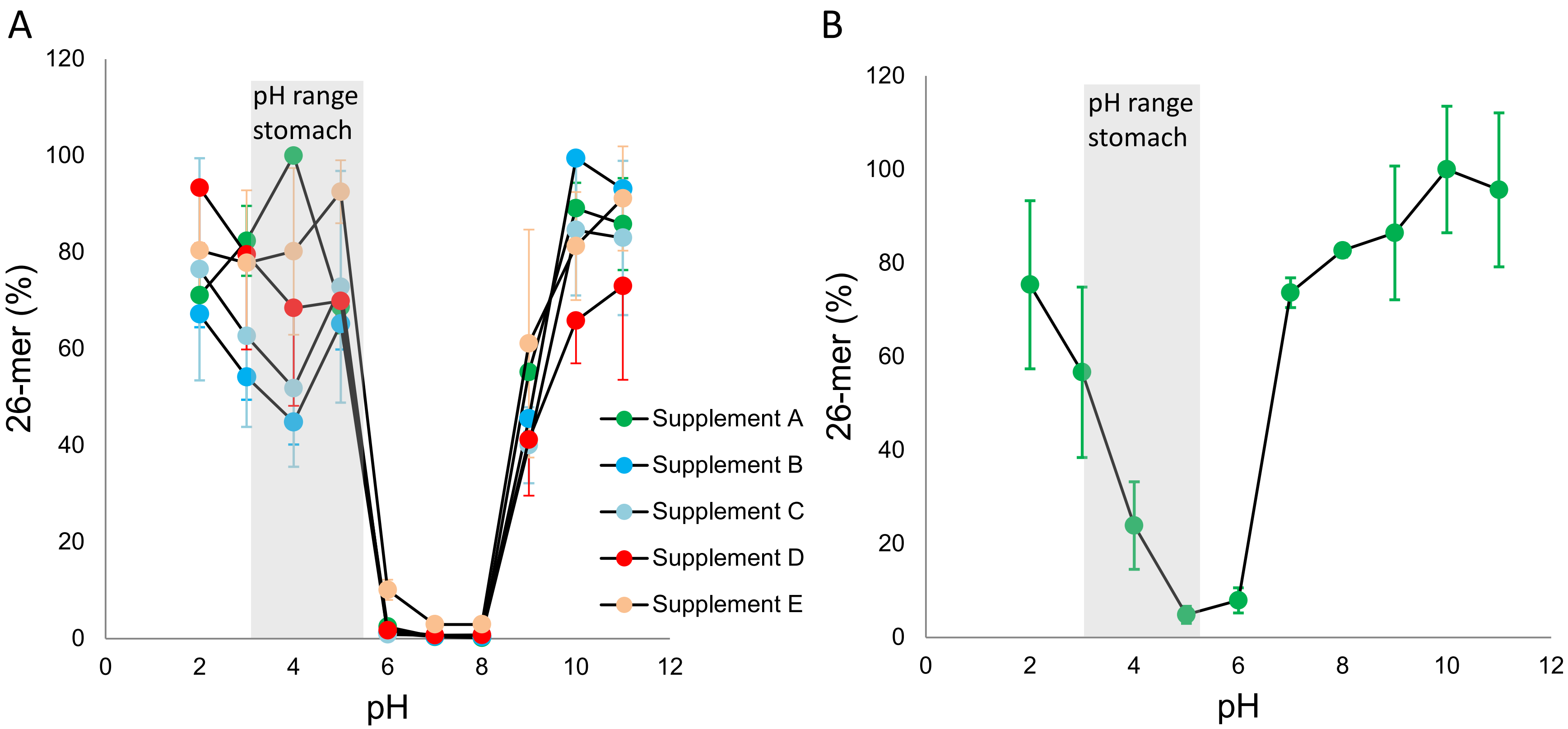

S3 Fig. Determination of pH optimum of digestive enzyme supplements using 1 capsule equivalent (A) and AN-PEP using 1/100 capsule equivalent as control (B). Supplements were incubated for 30 minutes at all the pHs 2 to 11 with 26-mer gluten peptide. The decrease of intact 26-mer (m/z 1049 (3+)) was monitored by mass spectrometry and taken as measure for activity. Note that in the case of supplements, the decrease of 26-mer does not reflect degradation of epitopes, but only exoprotease activity removing one to two amino acids at most from the N-terminus (Fig. 2, and S4 and S5 Figs). Error bars in A and B represent standard deviation for triplicate measurements.
