# Supplementary material for: Ineffective Degradation of Immunogenic Gluten Epitopes by Currently Available Digestive Enzyme Supplements
Source: PLoS One. 2015 Jun 1;10(6):e0128065. doi: 10.1371/journal.pone.0128065 (PMC4452362; doi:10.1371/journal.pone.0128065)
Supplement: S4 Fig — 26-mer peptide was incubated for 30 min with 1/100 capsule equivalent of AN-PEP (A) or with 1 capsule equivalent of Supplement A (B) at 10 different pHs 2–11. A, The peptides with m/z 504 (1+), 616 (1+) and 857 (1+) are short breakdown products derived from the 26-mer; a peptide with less than 8 amino acids is too short to contain an epitope. The small amounts of 679 (2+) and 1154 (2+) peptides disappear after prolonged treatment. B, The peptides with m/z 963 (3+) and 1001 (3+) still contain all three epitopes of the 26-mer 1049 (3+). Degradation patterns of the other 4 enzyme supplements are very similar to that of Supplement A. Epitope-containing peptides are marked with a dot (epitopes are underlined). Because of ammonia adduct formation, peptides resolve into several closely spaced peaks. The m/z values in the spectra correspond to the most intense species; the m/z shown below correspond to the monoisotopic mass. (PDF) [file pone.0128065.s004.pdf]

S4 Fig. Degradation of 26-mer by AN-PEP and digestive enzyme supplement. 26-mer peptide was incubated for 30 min with 1/100 capsule equivalent of AN-PEP (A) or with 1 capsule equivalent of Supplement A (B) at 10 different pHs 2-11. **A,** The peptides with m/z 504 (1+), 616 (1+) and 857 (1+) are short breakdown products derived from the 26-mer; a peptide with less than 8 amino acids is too short to contain an epitope. The small amounts of 679 (2+) and 1154 (2+) peptides disappear after prolonged treatment with AN-PEP. **B,** The peptides with m/z 963 (3+) and 1001 (3+) still contain all three epitopes of the 26-mer 1049 (3+). Degradation patterns of the other 4 enzyme supplements are very similar to that of Supplement A. Epitope-containing peptides are marked with a dot (epitopes are underlined). Because of ammonia adduct formation, peptides resolve into several closely spaced peaks. The m/z values in the spectra correspond to the most intense species; the m/z next to sequences correspond to the monoisotopic mass.

S4A Fig. Degradation of 26-mer by AN-PEP.

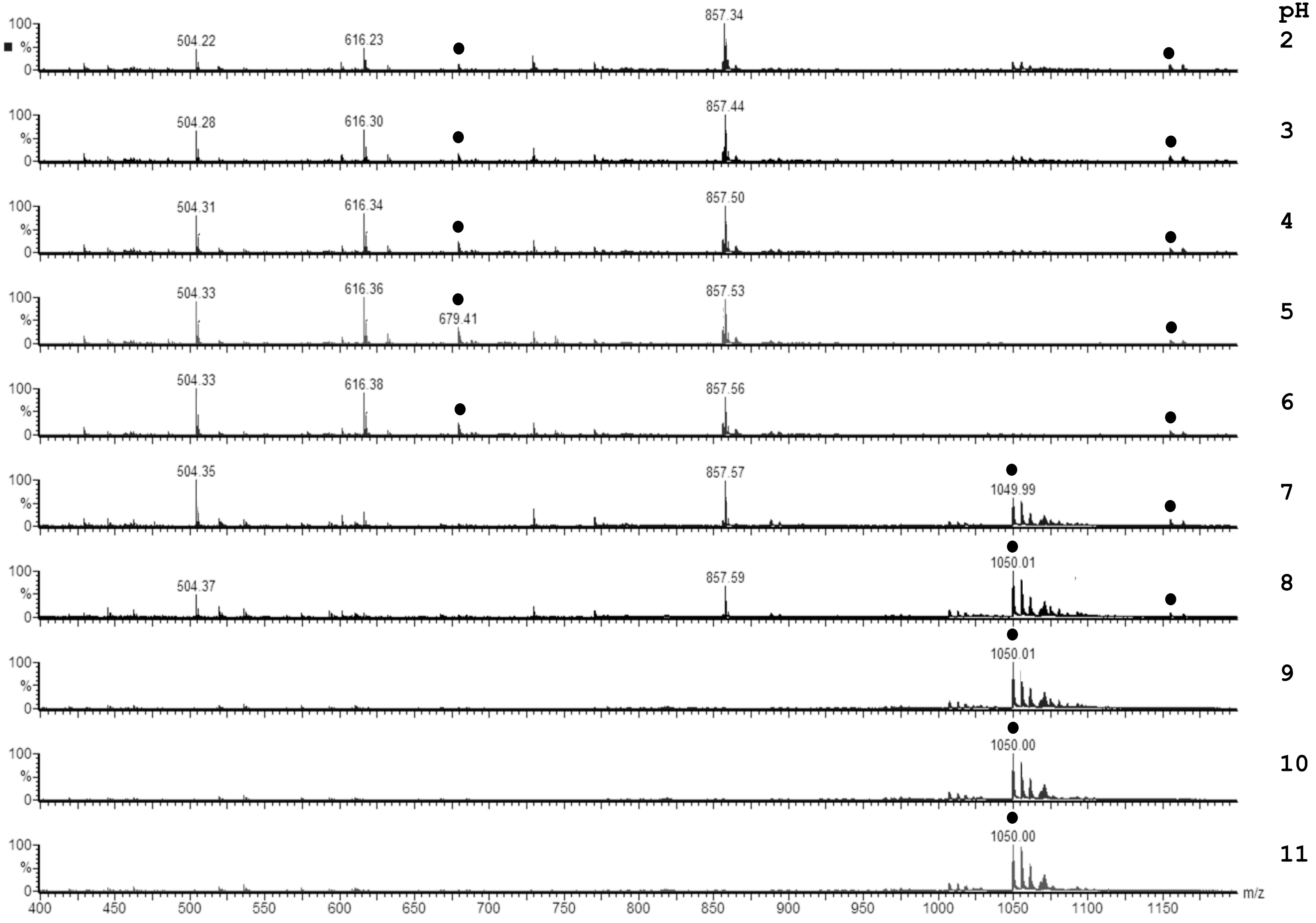

m/z 1049(3+) 26-mer	FLQP**QQPFPQQPQQPYPQQPQQPFPQ**
m/z 1154(2+)	-------FP**QQPQQPYPQQPQQPFPQ**
m/z 679(2+)	---------------YP**QQPQQPFPQ**

S4B Fig. Degradation of 26-mer by digestive enzyme Supplement A.

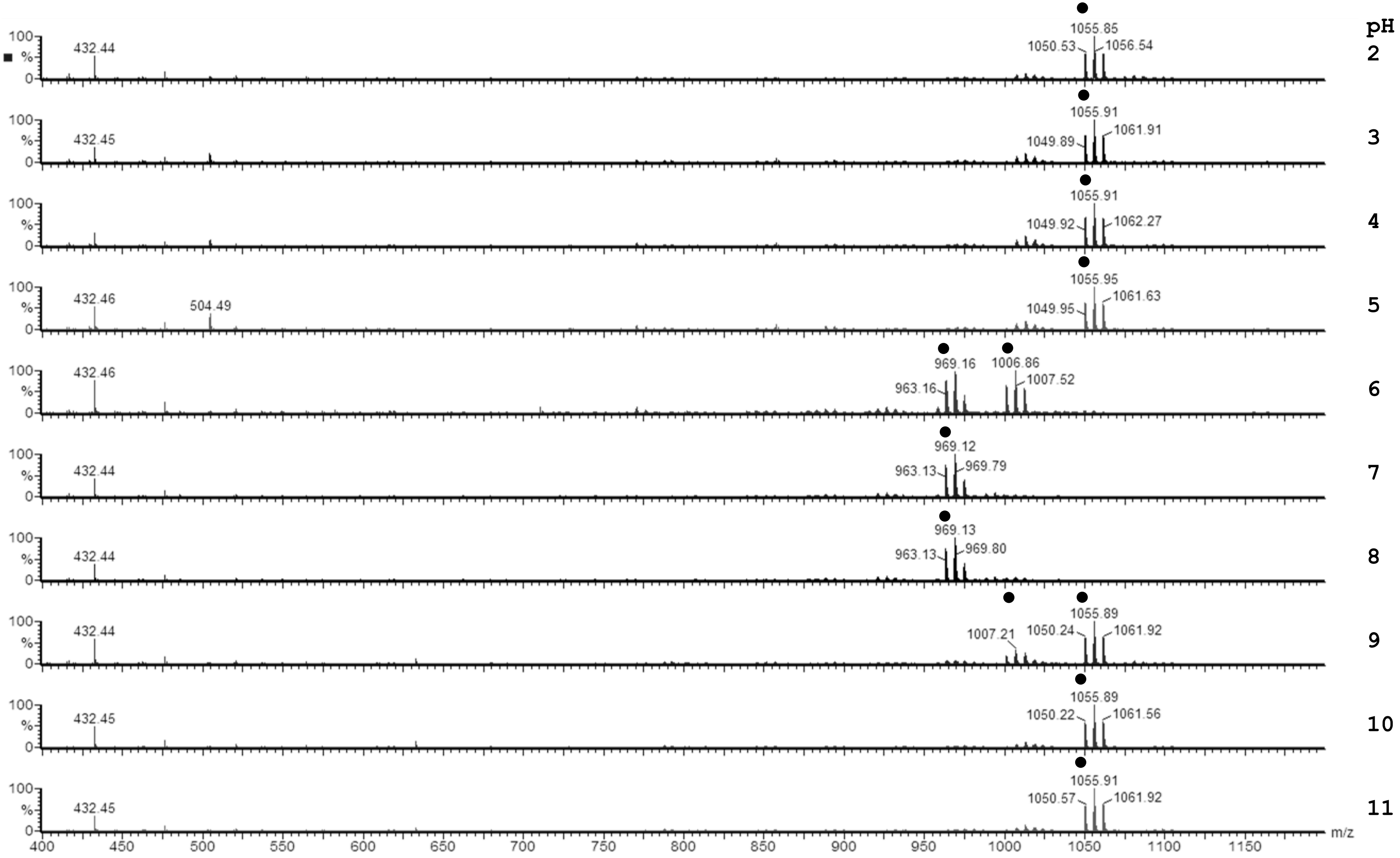

m/z 1049(3+)	26-mer		FLQP**<u>QQPFPQQPQQPYPQQPQQPFPQ</u>**
m/z 1001(3+)	26-mer-F		-LQP**<u>QQPFPQQPQQPYPQQPQQPFPQ</u>**
m/z 963(3+)	26-mer-FL		--QP**<u>QQPFPQQPQQPYPQQPQQPFPQ</u>**
