# Supplementary material for: Ineffective Degradation of Immunogenic Gluten Epitopes by Currently Available Digestive Enzyme Supplements
Source: PLoS One. 2015 Jun 1;10(6):e0128065. doi: 10.1371/journal.pone.0128065 (PMC4452362; doi:10.1371/journal.pone.0128065)
Supplement: S5 Fig — 33-mer peptide was incubated for 30 min with 1 capsule equivalent of digestive enzyme supplement or 1/100 capsule equivalent of AN-PEP at pH 2.0, 5.0 and 7.0 as shown. Peptides were analyzed by LC-MS as detailed in S2 Text Methods. Peptides containing epitopes are marked with a dot. The small residual amounts of 33-mer and 33-mer-LQLQP m/z 1111 (3+) in the case of AN-PEP disappear after prolonged treatment. Because of ammonia adduct formation, peptides resolve into several closely spaced peaks (see also S4 Fig). Degradation patterns of the other 4 digestive enzyme supplements are very similar to that of Supplement D. (PDF) [file pone.0128065.s005.pdf]

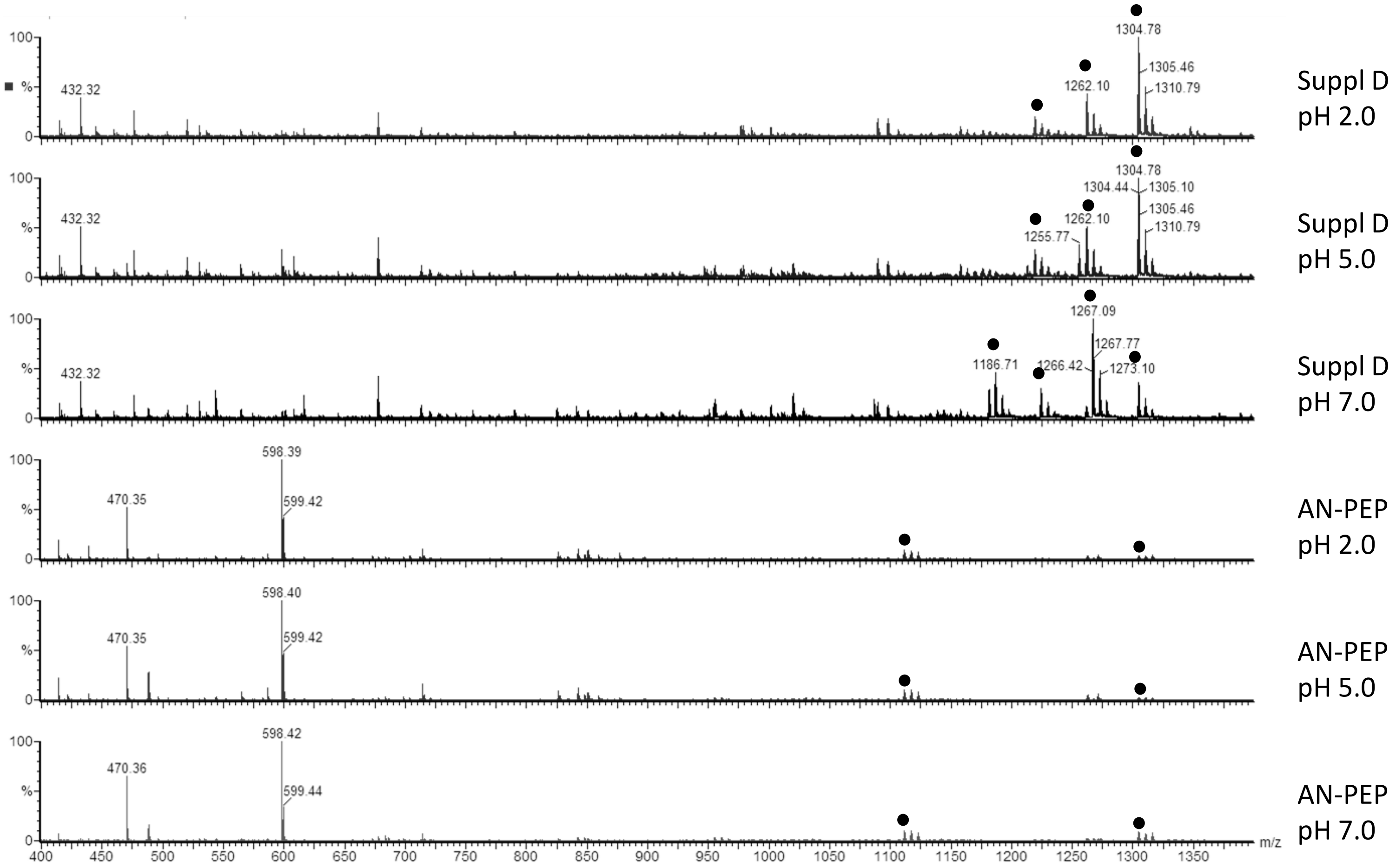

m/z 1304(3+) 33-mer LQLQ**PFPQPQLPYPQPQLPYPQPQLPYPQ**PQPF

m/z 1267(3+) 33-mer-L -QLQ**PFPQPQLPYPQPQLPYPQPQLPYPQ**PQPF

m/z 1224(3+) 33-mer-LQ --LQ**PFPQPQLPYPQPQLPYPQPQLPYPQ**PQPF

m/z 1187(3+) 33-mer-LQL ---Q**PFPQPQLPYPQPQLPYPQPQLPYPQ**PQPF

m/z 1111(3+) 33-mer-LQLQP -----F**PQPQLPYPQPQLPYPQPQLPYPQ**PQPF

S5 Fig. Degradation of 33-mer by digestive enzyme supplement D and AN-PEP. 33-mer peptide was incubated for 30 min with 1 capsule equivalent of digestive enzyme supplement or 1/100 capsule equivalent of AN-PEP at pH 2.0, 5.0 and 7.0 as shown. Peptides were analyzed by LC-MS as detailed in S2 Text Methods. Peptides containing epitopes are marked with a dot. The small residual amounts of 33-mer and 33-mer-LQLQP m/z 1111 (3+) in the case of AN-PEP disappear after prolonged treatment. Because of ammonia adduct formation, peptides resolve into several closely spaced peaks (see also S4 Fig.). Degradation patterns of the other 4 digestive enzyme supplements are very similar to that of Supplement D.
