# Supplementary material for: Ineffective Degradation of Immunogenic Gluten Epitopes by Currently Available Digestive Enzyme Supplements
Source: PLoS One. 2015 Jun 1;10(6):e0128065. doi: 10.1371/journal.pone.0128065 (PMC4452362; doi:10.1371/journal.pone.0128065)
Supplement: S1 Table — *AN-PEP is at present not commercially available in the form of a capsule; 275 mg is the intended capsule content; an amount of 100 ng AN-PEP compares to 1/100 capsule equivalent in the downscaled (27,500 x) assay. Capsule equivalents for digestive enzyme supplements were corrected for capsule content using a x b/c, where a = 100 ng, the optimal amount AN-PEP; b is the capsule content of enzyme supplements in mg; and c = 275 mg, the intended capsule content for AN-PEP (PDF) [file pone.0128065.s006.pdf]

S1 Table. Calculation of capsule equivalents

Digestive enzyme	Capsule content (mg)	1/100 capsule equivalent (ng)	1/10 capsule equivalent (µg)	1 capsule equivalent (µg)	10 capsule equivalent (µg)
supplement A	695	253	2.53	25.3	253
supplement B	335	122	1.22	12.2	122
supplement C	465	169	1.69	16.9	169
supplement D	395	144	1.44	14.4	144
supplement E	425	155	1.55	15.5	155
AN-PEP	275*	100	1.00	10.0	100

*AN-PEP is at present not commercially available in the form of a capsule; 275 mg is the intended capsule content; an amount of 100 ng AN-PEP compares to 1/100 capsule equivalent in the downscaled (27,500 x) assay.

Capsule equivalents for digestive enzyme supplements were corrected for capsule content using a x b/c, where a=100 ng, the optimal amount AN-PEP; b is the capsule content of enzyme supplements in mg; and c=275 mg, the intended capsule content for AN-PEP
