# Supplementary material for: Ineffective Degradation of Immunogenic Gluten Epitopes by Currently Available Digestive Enzyme Supplements
Source: PLoS One. 2015 Jun 1;10(6):e0128065. doi: 10.1371/journal.pone.0128065 (PMC4452362; doi:10.1371/journal.pone.0128065)
Supplement: S1 Text — (PDF) [file pone.0128065.s007.pdf]

S1 Text. Formulation of selected enzyme supplements

Supplement A: DPPIV (90 000 HUT), Protease (10 000 HUT)[**32,700 HUT**], Pepsin

Supplement B: DPPIV 100mg, Protease (30 000 HUT)[**70,400 HUT**]

Supplement C: DPPIV/Protease blend (140 000 HUT)

Supplement D: DPPIV (1000 DPPU), Protease (90 000 HUT)[**115,000 HUT**]

Supplement E: DPPIV (500 DPPU), Protease (40 000 HUT), Peptidase (20 000 HUT)[**34,400 HUT**], ANPEP (6000 HUT)

In bold and in brackets is HUT activity/capsule as measured in our assays.
Typically recommended dosages is 1-2 capsules prior to a meal.
Next to proteases, all supplements also contain one or more carbohydrate degrading enzymes, like amylase.
Activity numbers are rounded.
