# Supplementary material for: Ineffective Degradation of Immunogenic Gluten Epitopes by Currently Available Digestive Enzyme Supplements
Source: PLoS One. 2015 Jun 1;10(6):e0128065. doi: 10.1371/journal.pone.0128065 (PMC4452362; doi:10.1371/journal.pone.0128065)
Supplement: S2 Text — (PDF) [file pone.0128065.s008.pdf]

S2 Text. Supporting Information Methods

Characterization of supplement composition

Digestive enzyme supplements were dissolved in 20 mM ammonium acetate pH 6.0 at a concentration of 10 mg/ml and insoluble material removed by centrifugation. 2 µl of the clear solution was subjected to denaturation with 10% trichloroacetic acid, followed by neutralization and denaturation with 1 M NaOH, both for 15 min at ambient temperature. After neutralization to pH 8.4, proteins were reduced by incubation with 3 mM DTT for 20 min at 54°C, alkylated by incubation with 10 mM iodoacetamide for 20 min at ambient temperature and purified by filtration (with 3 washes of 25 mM NH4HCO3 pH 8.4) on 10 kDa Microcon filters (Millipore) to remove small (<10 kDa) impurities that may disturb mass spectrometric identification of the proteins [1]. The protein residue on top of the filter was dissolved in 200 µl of the same buffer and subjected to overnight digestion at ambient temperature with 2 µg trypsin (Worthington Enzymes). The filter was then centrifuged to collect tryptic peptides which pass the 10 kDa filter leaving behind undigested protein and trypsin on top of the filter. The filtrate was lyophilized, dissolved in 95/3/0.1 v/v/v water/acetonitrile/formic acid and subsequently analyzed by on-line nanoHPLC MS/MS using an 1100 HPLC system (Agilent Technologies), as previously described [2]. Peptides were trapped at 10 µl/min on a 15-mm column (100-µm ID; ReproSil-Pur C18-AQ, 3 µm, Dr. Maisch GmbH) and eluted to a 200 mm column (50-µm ID; ReproSil-Pur C18-AQ, 3 µm) at 150 nl/min. All columns were packed in house. The column was developed with a 30-min gradient from 0 to 30% acetonitrile in 0.1% formic acid. The end of the nanoLC column was drawn to a tip (ID ~5 µm), from which the eluent was sprayed into a 7-tesla LTQ-FT Ultra mass spectrometer (Thermo Fisher Scientific). The mass spectrometer was operated in data-dependent mode, automatically switching between MS and MS/MS acquisition. Full scan MS spectra were acquired in the FT-ICR with a resolution of 25,000 at a target value of 3,000,000. The two most intense ions were then isolated for accurate mass measurements by a selected ion monitoring scan in FT-ICR with a resolution of 50,000 at a target accumulation value of 50,000. Selected ions were fragmented in the linear ion trap using collision-induced dissociation at a target value of 10,000. Raw files were converted to peak lists (mgf) using Proteome Discoverer v.1.4 (Thermo Fisher Scientific), then submitted to the Uniprot database (532,146 entries) using Mascot v2.2.04 (www.matrixscience.com) for protein identification.

Mascot searches were performed with 2 ppm and 0.5 Da deviation for precursor and fragment mass, respectively, and the Mascot data files visualized using Scaffold v4.0.7 software (www.proteomesoftware.com).

For MS analysis of selected SDS PAGE bands, bands were reduced with dithiothreitol, alkylated using iodoacetamide and digested with trypsin using a Proteineer DP digestion robot (Bruker). The tryptic peptides were extracted from the gel, lyophilized, dissolved in 95/3/0.1 v/v/v water/acetonitrile/formic acid and subsequently analyzed by on-line nanoLC MS/MS as described above.

Mass spectrometric analysis of epitope breakdown

All protease preparations were first purified by filtration on 10 kDa Microcon filters (Millipore) to remove small (<10 kDa) impurities that may disturb mass spectrometric analysis of gluten epitopes and degradation products. The five digestive enzyme preparations and pure enzyme preparations (AN-PEP, DPPIV and LAP) were dissolved in 20 mM ammonium acetate pH 6.0 to a concentration of 10 mg/ml and kept cold throughout the procedure. 40-µg protein aliquots were diluted to 100 µl with 20 mM buffer pH 6.0, applied on the 10 kDa filter and centrifuged for 15 min at 14000 x g. The protein residing on top of the filter (residue) was then washed three times with 100 µl 20 mM ammonium acetate buffer pH 6.0. The protein residue was dissolved in the same buffer. 100 ng of purified AN-PEP or purified digestive enzyme supplement (amount calculated as described below) was diluted in 200 µl of the appropriate buffer pH 2.0-11.0. To cover the complete pH range of 2.0 to 11.0 in 1.0 pH intervals, volatile buffer solutions were prepared starting with ammonium bicarbonate at a concentration of 100 mM. pH was adjusted with either acetic acid (and hydrochloric acid to reach pH 2.0) or ammonia. Reaction mixtures were prepared at 0°C and consisted of 40 µg peptide substrate (26-mer or 33-mer peptide) and 100 ng AN-PEP (this corresponds to 1/100 capsule equivalent), or 1 capsule equivalent of digestive enzyme supplement in a final volume of 200 µl 100 mM buffer pH 2.0 to 11.0. Digestion was started by incubation at 37 °C. After 0, 10, 30, 60 and 120 minutes, 40-µl aliquots were removed and immediately chilled on ice-water to inhibit further digestion. Aliquots were directly processed by filtration on pre-washed and pre-chilled 30 kDa Microcon filters (Millipore).

The filtrate, containing the 26-mer or 33-mer substrate and their degradation products, was freeze-dried and stored at -20 °C. Peptides were dissolved in 0.1% formic acid and analyzed by on-line nano LC-electrospray mass spectrometry on a Q-TOF Ultima (Micromass) using a similar system as described above, but without the analytical column. After washing on the pre-column, peptides were block-eluted into the mass spectrometer. Selected peptides were subjected to MS/MS and their sequences determined by a combination of mass matching within the precursor peptide, using Masslynx version 4.1 and manual interpretation.

Calculation of capsule equivalents

When considering 275 mg AN-PEP as the content of one capsule, then an amount of 100 ng AN-PEP compares to 1/100 capsule equivalent in the downscaled assay, where a dilution factor of 27,500 was applied to allow accurate measurements; 1/100 x 275 mg / 27500 = 0.0001 mg ≡ 100 ng. Capsule equivalents for digestive enzyme supplements were corrected for capsule content using a x b/c, where a=100 ng, the optimal amount AN-PEP; b is the capsule content of enzyme supplements in mg; and c=275 mg, the intended capsule content for AN-PEP (see also S1 Table). Under conditions that AN-PEP degraded 90% of the 26-mer, none of the digestive enzyme supplements (at 1/100 capsule equivalent) displayed any activity (results not shown).

[1] Wiśniewski JR, Zougman A, Nagaraj N, Mann M. Universal sample preparation method for proteome analysis. Nat Methods 2009; 6: 359–62.
[2] Hassan C, Kester MG, de Ru AH, Hombrink P, Drijfhout JW, Nijveen H, et al. The human leukocyte antigen-presented ligandome of B lymphocytes. Mol Cell Proteomics 2013; 12:1829-43.
